# A time trade-off study to determine health-state utilities of transplant recipients with refractory cytomegalovirus infection with or without resistance

**DOI:** 10.1186/s12955-024-02239-w

**Published:** 2024-03-06

**Authors:** Waqas Ahmed, Louise Longworth, Yemi Oluboyede, Peter Cain, Stacey L. Amorosi, Sarah Hill, Ishan Hirji

**Affiliations:** 1 PHMR Ltd, London, UK; 2 Takeda UK Ltd, London, UK; 3 Takeda Development Center Americas, Inc, 300 Shire Way, Lexington, MA 02421 USA

**Keywords:** Cytomegalovirus infections, Quality of life, Survey and questionnaires, Time trade off, Utility

## Abstract

**Background:**

Health-state utility values (HSUVs) for post-transplant refractory cytomegalovirus (CMV) infection (with or without resistance [R/R]) were determined using a time trade-off (TTO) survey completed by 1,020 members of the UK general public.

**Methods:**

Existing literature and qualitative interviews with clinicians experienced in treating R/R CMV were used to develop initial draft vignettes of health states. The vignettes were refined to describe three clinical states of R/R CMV: clinically significant and symptomatic (CS-symptomatic CMV); clinically significant and asymptomatic (CS-asymptomatic CMV); and non-clinically significant (non-CS CMV). Each clinical state was valued independently and combined with three events of interest: graft-versus-host disease; kidney graft loss; and lung graft loss to generate twelve vignettes. The final vignettes were evaluated by a sample of the UK general public using an online TTO survey. Exclusion criteria were applied to the final data to ensure that responses included in the analysis met pre-defined quality control criteria.

**Results:**

Overall, 738 participants met the inclusion criteria and were included in the analysis. The sample was representative of the UK general population in terms of age and sex. Non-CS CMV had the highest mean HSUV (95% confidence interval) (0.815 [0.791, 0.839]), followed by CS-asymptomatic CMV (0.635 [0.602, 0.669]), and CS-symptomatic CMV (0.443 [0.404, 0.482]). CS-symptomatic CMV with lung graft loss had the lowest mean HSUV (0.289), with none of the health states considered on average worse than dead.

**Conclusions:**

Post transplant R/R CMV has substantial impact on the health-related quality of life of patients. The utility values obtained in this study may be used to support economic evaluations of therapies for R/R CMV infection.

**Supplementary Information:**

The online version contains supplementary material available at 10.1186/s12955-024-02239-w.

## Background

Cytomegalovirus (CMV) infection and disease following solid organ transplant (SOT) or hematopoietic stem cell transplant (HSCT) are common complications that may threaten transplant viability and increase the risk of mortality and morbidity [1, 2]. CMV infection may occur due to transmission from the transplanted organ, reactivation of a latent infection, or after a primary infection in seronegative patients [3]. The incidence of CMV may vary according to the type of transplant, serological match between donor and recipient, immunosuppressive drugs, and interference of additional illness risk factors [3]. Clinical manifestations of CMV infection post-transplant range from fever, malaise, leukopenia, thrombocytopenia, elevated liver enzymes, and other severe complications, such as retinitis, pneumonia, hepatitis, and encephalitis [3].

Drugs that have been used conventionally for the management of CMV in transplant recipients include intravenous (IV) ganciclovir, oral valganciclovir, IV foscarnet, and IV cidofovir [1, 3]. Letermovir is indicated as a prophylactic agent to prevent CMV reactivation and disease in adult recipients of allogeneic HSCT who are seropositive for CMV [4]. Some patients fail to respond to conventional antiviral therapies and develop refractory CMV infection (with or without resistance [R/R]) [5]. A systematic review of observational studies in the US and Europe reported the incidence of resistant CMV infection among SOT recipients to be between 0.6% and 13.8% and that in HSCT recipients to be 1.8%–4.1% [6]. Foscarnet and cidofovir may be used as second-line options to treat refractory or ganciclovir-resistant CMV [7]. However, the use of these drugs is associated with significant toxicities, including renal toxicity and electrolyte imbalance [1]. Maribavir is an orally bioavailable anti-CMV agent used for the treatment of R/R CMV infection or disease in transplant recipients [8]. Results from a phase 3 study (SOLSTICE; NCT03869892) demonstrated that patients treated with maribavir had improved CMV clearance and fewer treatment discontinuations due to treatment-emergent adverse events than patients treated with investigator-assigned therapy (valganciclovir, ganciclovir, foscarnet, or cidofovir) [8].

Post-transplant CMV infection is associated with considerable healthcare resource utilization and costs [9]. Studies have also shown that post-transplant CMV infection has a detrimental impact on the health-related quality of life (HRQoL) of allogeneic HSCT [10] and SOT [11] recipients. Further, many health technology assessments agencies, including the National Institute for Health and Care Excellence (NICE) in the UK, require health-related utility data to inform economic evaluations of treatments and to calculate quality adjusted life years [12]. However, there is limited evidence on the impact of R/R CMV on the HRQoL of transplant recipients, as well as limited health-related utility data for this population.

EQ-5D, the instrument of choice by NICE, uses a standardized health state descriptive system consisting of five dimensions: mobility, self-care, usual activities, pain/discomfort and anxiety/depression [13, 14]. SOLSTICE was the first trial to assess EQ-5D in patients with R/R CMV, and no meaningful differences in HRQoL were observed by treatment arm (data unpublished), potentially due to heterogeneity of the severity of disease and complexities of the patient population enrolled. However, differences were observed when the EQ-5D data were re-analyzed by viremia clearance at 8 weeks (SOLSTICE primary endpoint), providing base case utility data in a cost-effectiveness model developed from a UK perspective [15].

The use of vignettes is recommended as an alternative by NICE when EQ-5D data are either unsuitable or unavailable [16]. CMV can have diverse and complex impacts on the HRQoL of post-transplant patients [10, 11], and EQ-5D-5L data captured during the SOLISTICE trial may not have adequately captured all of the relevant effects on patient HRQoL of R/R CMV, its complications, and its treatment. Therefore, there were limited EQ-5D data available for all the health states required for the cost-effectivenes model. The present vignette study aimed to elicit additional health-related utility data associated with R/R CMV infection in SOT and HSCT recipients using the time trade-off (TTO) technique to value a series of health-state vignettes. TTO involves constructing brief health-state descriptions of the typical experiences of patients with a particular condition, which are then valued to elicit health-state utility values (HSUVs) for each corresponding health state [16]. Furthermore, vignettes can capture all the relevant effects of R/R CMV on patient HRQoL, its complications, and treatment [17].

## Methods

### Vignette development

Initial health-state vignettes describing symptoms, conventional anti-CMV treatments, and effects of R/R CMV and its common complications on patient HRQoL post-SOT or HSCT were developed. Health states were constructed using data from the existing literature and information from patient-centered websites [18–21]. The draft vignettes were further refined based on the findings of interviews with five UK-based clinicians experienced in treating R/R CMV in either SOT or HSCT recipients. The clinical experience of these clinicians is provided in Supplementary Table 1. The clinicians were asked to share their perceptions of the HRQoL and daily experiences of patients with post-transplant R/R CMV. The vignettes described three clinical health states: clinically significant and symptomatic CMV (CS-symptomatic CMV), clinically significant and asymptomatic CMV (CS-asymptomatic CMV), and non-clinically significant CMV (non-CS CMV). Consistent with the pivotal SOLSTICE trial [8], CS-CMV was defined as CMV DNA above the lower limit of quantification (LLOQ; screening value ≥ 137 IU/mL in 2 consecutive tests separated by ≥ 1 day determined by quantitative polymerase chain reaction), whilst non-CS CMV was defined as CMV DNA below the LLOQ or CMV DNA above the LLOQ not requiring treatment. Each clinical health state was considered as a stand-alone vignette and was also combined with health-state descriptions for each of the three included events of interest. These events of interest were graft-versus-host disease (GvHD) as a post-HSCT complication, kidney graft loss (GL) as a post-SOT complication, and lung GL as a post-SOT complication. Kidney GL was selected because kidney transplant is the most common type of transplant surgery in the UK [22]. Lung GL was selected because lung transplants are in high demand in the UK and recipients have a lower median survival rate compared with other SOT [22, 23].

A thematic analysis of the transcripts of interviews with clinicians was conducted. The qualitative data were analyzed using MAXQDA software (20.2.1; VERBI Software, Berlin, Germany). The findings from the qualitative analysis informed amendments of the draft vignettes to generate the final set of vignettes for use in the TTO survey (Supplementary Tables 2–13).

### TTO analysis and survey administration

**Fig. 1:**
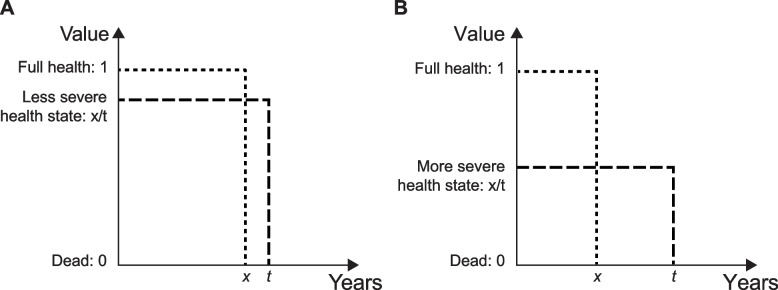
A conceptual illustration of a composite TTO task for health states considered better than dead on a scale where 0 represents a state as bad as being dead and 1 represents full health. The respondent is asked to choose between 2 lives: *x* years in full health, or *t* years in an impaired health state. **A** An example of a less severe health state that would be given a relatively high value. **B** An example of a more severe health state that would be given a relatively low value

The TTO methodology applied in this study was based on an international protocol used to value EQ-5D-5L health states [14, 24]. A composite TTO framework, comprising a conventional and lead-time TTO, was used to allow respondents to assign positive or negative utility values to health states [25] on a scale where 0 represents a state as bad as being dead and 1 represents full health (Fig. 1). TTO tasks involved determining the length of life in full health that a respondent was willing to sacrifice to avoid living in an impaired state of health for a fixed time (e.g., 10 years). An iterative process was undertaken in which the amount of time in full health was altered (either increased or decreased based on the respondent’s prior response) until a point of preferential indifference was reached between living in an impaired state of health for a fixed time and living in full health for an equal or lesser time. At this point, the respondent’s utility value for the health state was calculated based on the length of time in full health that the respondent perceived to be equal to the fixed time in the impaired health state. The utility value scale had anchors at zero, representing dead, and one, representing full health; negative utility values represented states of health considered worse than being dead.

The TTO survey was developed and hosted using an online platform and administered to a panel of 1,020 anonymized respondents (aged ≥ 18 years) from the UK general public between the 6th and 13th of October 2021. To minimize the risk of potential cognitive overload, participants were each asked to value nine of the twelve vignette health states. All respondents valued the three clinical health-state vignettes CS-symptomatic CMV, CS-asymptomatic CMV, non-CS CMV, both with and without GvHD (*n* = 6 health states valued by all participants). The study sample was randomized to value either the lung GL or kidney GL health states in approximately equal proportions. Prior to undertaking the main TTO valuation tasks, participants were presented with an instructional video and were required to complete a practice TTO task valuing a mild generic health state unrelated to CMV. During the TTO valuation tasks, participants were able to return to a previous step to adjust their responses if required. To ensure the study design and content were suitable, a soft launch was conducted with approximately 10% of the overall respondent sample.

Descriptive statistics were used to analyze the TTO data; utilities were calculated and the mean, median, standard error (SE), and 95% confidence interval (CI) utility values for each health state were presented. A regression analysis using a linear regression model with ulitity as the dependent variable was conducted to assess potential differences across sugroups of participant background characteristics for each health state. The data were analyzed using Stata version 15.1 (Stata Corporation, Stata Corporation, College Station, TX, USA).

### Quality control criteria

Quality control criteria were used to remove low quality responses from the data analysis. These criteria included assigning all health states the same utility value, assigning a lower utility value to the least severe clinical health state (non-CS CMV) than the more severe clinical health states (CS-symptomatic CMV and CS-asymptomatic CMV), and completing the online TTO survey faster than 50% of the median completion time for the overall sample (i.e., 10 min and 25 s). These three criteria were adapted from quality controls implemented in the UK valuation study in which a value set was produced to support the use of EQ‐5D‐5L data in decision‐making [26].

## Results

### Respondent demographics

Among the 1,020 respondents from the UK general public who valued the vignettes via the online TTO survey, 738 met the quality control criteria and were included in the analysis (Table 1). The 738 respondents were largely representative of the UK general population with regards to age and sex [27]. However, respondents who were aged 65 to 74 years were slightly over-represented. Most respondents were in the age group of 45 to 54 years old, had obtained college or university education, and reported a “good” level of current health (Table 2). The mean and median survey completion times were 26 min and 31 s and 20 min and 50 s, respectively.

**Table 1:** Summary of quality control criteria utilized to ensure quality responses

Criteria	Respondent sample (*N* = 1,020)	%
**Summary of excluded responses**		
Assigned same value to all health states	139	13.6
Valued non-CS CMV lower than CS-symptomatic CMV and CS-asymptomatic CMV	96	9.4
Completed the survey(s) faster than 10 min and 25 s	78	7.6
**Total number of respondents excluded from analysis**	282	27.6
**Total number of respondents included in analysis**	738	72.4

*CMV* cytomegalovirus, *CS-asymptomatic CMV* clinically significant and asymptomatic cytomegalovirus, *CS-symptomatic CMV* clinically significant and symptomatic cytomegalovirus, *non-CS CMV* non-clinically significant cytomegalovirus

**Table 2:** Summary of respondent characteristics

**Characteristic**	**Respondent sample (*N* = 738)**	**%**	**UK population (%)**^a^
**Sex**			
Male	341	46.2	51.4
Female	394	53.4	48.6
Prefer not to say	2	0.3	N/A
Other	1	0.1	N/A
**Age (years)**			
18–24	70	9.5	11.9
25–34	124	16.8	17.1
35–44	125	16.9	17.8
45–54	146	19.8	17.5
55–64	138	18.7	14.9
65–74	104	14.1	8.7
≥ 75	31	4.2	7.8
**Education**			
Primary school	5	0.7	N/A
Secondary school up to 16 years	166	22.5	N/A
Higher or secondary or further education(e.g., A levels, BTEC)	180	24.4	N/A
College or university	263	35.6	N/A
Post-graduate degree	122	16.5	N/A
Prefer not to say	2	0.3	N/A
**Current health**			
Very good	139	18.8	35.8
Good	330	44.7	43.1
Fair	225	30.5	15.7
Bad	29	3.9	4.3
Very bad	15	2.0	1.1

*ADHS* Adult Dental Health Survey 2009, *BTEC* Business and Technician Education Council, *N/A* not available

^a^Data for sex and age from 2011 census [27]; data for current health from 2009 ADHS survey [28]

### Results from the qualitative interviews of the clinicians

#### Clinically significant and symptomatic CMV (CS-symptomatic CMV)

According to the responses from the clinician interviews, diarrhea or colitis were the most common symptoms reported in patients with post-transplant R/R CS-symptomatic CMV. This condition was reported to reduce the usual activities of patients and had a negative impact on their mental health. With regards to treatment, the clinicians had a mixed response as to whether oral or IV therapy was typically used as first-line treatment, depending on the patient’s hospitalization status and symptomatic profile. Two of the five clinicians stated that, if the patient was experiencing diarrhea and nausea or vomiting, IV over oral therapy was preferred given that patients would be unlikely to be able to swallow tablets. Additionally, restrictions to patient mobility were made specific to those who would require long infusions, unlike for those who would receive oral treatment.

### Clinically significant and asymptomatic CMV / non-clinically significant CMV (CS-asymptomatic CMV / non-CS CMV)

Although patients with R/R CS-asymptomatic CMV had minor or no symptoms, three of the five clinicians reported that the HRQoL of these patients was affected by conventional anti-CMV treatment and its associated side effects. Most clinicians stated that oral treatment was used as the first-line therapy, and that patients with post-transplant R/R non-CS CMV did not typically experience any symptoms. Two of the five clinicians reported that these patients were usually anxious about worsening or recurrence of their CMV infection.

### GvHD and graft loss

The most common symptoms reported in HSCT recipients with R/R CMV and GvHD were skin rash, diarrhea, and liver-related symptoms. Patients could develop GvHD even if they did not experience any CMV-related symptoms or if their CMV was not clinically significant. Similarly, with regards to post-SOT R/R CMV, all five clinicians reported that graft loss could develop even if patients did not experience any CMV-related symptoms or if their CMV was not clinically significant. Based on the responses of all clinicians experienced in treating post-SOT R/R CMV, it was confirmed that the symptoms and impact of experiencing graft loss were dependent on the type of solid organ that patients received (e.g., lung or kidney).

### TTO results

Responses to the TTO questions for each health state were converted into utilities and are summarized in Table 3. The mean HSUVs followed a logical pattern, with utility values decreasing as health states became more severe. Of the three clinical health states, non-CS CMV had the highest mean HSUV of 0.815 (95% CI: 0.791, 0.839) followed by CS-asymptomatic CMV (0.635 [95% CI: 0.602, 0.669]) and CS-symptomatic CMV (0.443 [95% CI: 0.404, 0.482]). CS-symptomatic CMV with lung GL had the lowest mean HSUV (0.289 [95% CI: 0.226, 0.352]). Among the three clinical health states, the lowest values were reported for health states including lung GL (CS-symptomatic CMV with lung GL: 0.289 [0.226, 0.352], CS-asymptomatic CMV with lung GL: 0.356 [0.290, 0.422], non-CS CMV with lung GL: 0.376 [0.312, 0.440]); however, these health states also had the largest 95% CI. No health state was assigned a negative mean utility value, demonstrating that overall, the respondent sample considered all health states to be better than being dead. Respondents also considered GvHD (a post-HSCT-specific complication) to be less severe than both kidney GL and lung GL (post-SOT-specific complications), and lung GL to be more severe than kidney GL when clinical health states were kept constant.

**Table 3:** Summary of health state utility values

Health state category	Health state	Number of respondents valuing health state	Mean utility (95% CI)	Median utility (SE)
Clinical health states	CS-symptomatic CMV	738	0.443 (0.404, 0.482)	0.500 (0.020)
	CS-asymptomatic CMV	738	0.635 (0.602, 0.669)	0.800 (0.017)
	Non-CS CMV	738	0.815 (0.791, 0.839)	0.975 (0.012)
Clinical health states with GvHD	CS-symptomatic CMV with GvHD	738	0.391 (0.350, 0.432)	0.500 (0.021)
	CS-asymptomatic CMV with GvHD	738	0.486 (0.446, 0.525)	0.625 (0.020)
	Non-CS CMV with GvHD	738	0.552 (0.514, 0.590)	0.700 (0.019)
Clinical health states with kidney GL	CS-symptomatic CMV with kidney GL	376	0.363 (0.303, 0.424)	0.500 (0.031)
	CS-asymptomatic CMV with kidney GL	376	0.470 (0.414, 0.526)	0.563 (0.028)
	Non-CS CMV with kidney GL	376	0.531 (0.480, 0.582)	0.650 (0.026)
Clinical health states with lung GL	CS-symptomatic CMV with lung GL	362	0.289 (0.226, 0.352)	0.450 (0.032)
	CS-asymptomatic CMV with lung GL	362	0.356 (0.290, 0.422)	0.500 (0.034)
	Non-CS CMV with lung GL	362	0.376 (0.312, 0.440)	0.500 (0.033)

*CMV* cytomegalosvirus, *CS-asymptomatic CMV* clinically significant and asymptomatic CMV, *CS-symptomatic CMV* clinically significant and symptomatic CMV, *GvHD* graft-versus-host disease, *GL* graft loss, *non-CS CMV* non-clinically significant CMV, *SD* standard deviation, *SE* standard error, *CI* confidence interval

### Regression analysis

Gender, age, education level, and survey completion time had a statistically significant impact on the variance of health state utility values although R^2^ values were low for all the models indicating that only a small amount of variation is being captured in the models (Table 4). Male participants assigned higher mean utility values to CS-symptomatic CMV with GvHD and lung GL, CS-asymptomatic CMV with kidney GL and lung GL, and non-CS CMV with lung GL than female participants and those responding ‘Other’ and ‘Prefer not to say’. Increased participant age had a small association with lower utility scores for all health states across lung GL. Participant education level had a marginally positive relationship with the mean utility value in CS-symptomatic CMV, CS-asymptomatic CMV, CS-asymptomatic CMV with GvHD and kidney GL, non-CS CMV, non-CS CMV with GvHD and kidney GL. Survey completion time had a small negative impact on the mean utility value of all health states except non-CS CMV.

**Table 4:** Results from a linear regression analyses^a^

	**Health state**	**Background characteristic**	**Coefficient**	**SE**	**R**^**2**^	**95% CI**
Clinical health states	CS-symptomatic CMV	Education level	0.053**	0.016	0.050	0.022, 0.084
		Completion time	− 0.061***	0.011		− 0.083, − 0.039
	CS-asymptomatic CMV	Education level	0.046**	0.014	0.019	0.018, 0.073
		Completion time	− 0.026*	0.010		− 0.046, − 0.006
	Non-CS CMV	Education level	0.045***	0.013	0.012	0.020, 0.071
Clinical health states with GvHD	CS-symptomatic CMV with GvHD	Gender	0.074*	0.036	0.063	0.004, 0.144
		Education level	0.049**	0.017		0.017, 0.082
		Completion time	− 0.076***	0.012		− 0.099, − 0.053
	CS-asymptomatic CMV with GvHD	Education level	0.044**	0.016	0.052	0.012, 0.075
		Completion time	− 0.060***	0.011		− 0.082, − 0.037
	Non-CS CMV with GvHD	Education level	0.055***	0.016	0.052	0.024, 0.085
		Completion time	− 0.057***	0.011		− 0.078, − 0.035
Clinical health states with kidney GL	CS-symptomatic CMV with kidney GL	Completion time	− 0.078***	0.018	0.064	− 0.113, − 0.043
	CS-asymptomatic CMV with kidney GL	Gender	0.111*	0.048	0.092	0.016, 0.206
		Education level	0.048*	0.022		0.004, 0.092
		Completion time	− 0.075***	0.017		− 0.107, − 0.042
	Non-CS CMV with kidney GL	Education level	0.060**	0.021	0.068	0.018, 0.101
		Completion time	− 0.058***	0.016		− 0.089, − 0.027
Clinical health states with lung GL	CS-symptomatic CMV with lung GL	Gender	0.154**	0.053	0.133	0.050, 0.259
		Age	− 0.036*	0.017		− 0.069, − 0.003
		Completion time	− 0.109***	0.017		− 0.142, − 0.075
	CS-asymptomatic CMV with lung GL	Gender	0.123*	0.055	0.112	0.015, 0.231
		Age	− 0.050**	0.017		− 0.084, − 0.016
		Completion time	− 0.095***	0.018		− 0.130, − 0.061
	Non-CS CMV with lung GL	Gender	0.125*	0.055	0.075	0.016, 0.233
		Age	− 0.048**	0.017		− 0.082, − 0.014
		Completion time	− 0.059**	0.018		− 0.094, − 0.024

*CMV* cytomegalosvirus, *CS-asymptomatic CMV* clinically significant and asymptomatic CMV, *CS-symptomatic CMV* clinically significant and symptomatic CMV, *GL* graft loss, *GvHD* graft-versus-host disease, *Non-CS CMV* non-clinically significant CMV, *SD* standard deviation, *SE* standard error, *CI* confidence interval

^a^Covariates included for each health state were gender, age, education level, current health, and completion time; only covariates that were statistically significant in each model are presented in table

^*^p < 0.05

^**^p < 0.01

^***^p < 0.001

## Discussion

Data on the HRQoL of transplant recipients with R/R CMV are limited. To the best of our knowledge, this is the first study that aimed to determine HSUVs for transplant recipients with R/R CMV to evaluate the impact of R/R CMV on patients’ HRQoL. Vignettes describing the symptoms, conventional anti-CMV treatments, and effects of R/R CMV across various severity levels, transplant types, and transplant complications were developed. Each vignette was evaluated by respondents from the general population in the UK using the composite TTO method. The TTO preference elicitation method is widely used to determine utility values of health outcomes or health states to inform economic evaluations [16].

Overall, the results from this study demonstrated that as the severity of the health states increased, the mean HSUVs reduced. The highest mean utility value was observed for non-CS CMV, while the lowest utility value was observed for CS-symptomatic CMV with lung GL. Lower utility values were also observed for combination health states compared with non-combination health states (i.e., a lower utility value was observed for CS-symptomatic CMV with GvHD than for CS-symptomatic CMV). This finding indicates that GvHD and graft loss can have a substantial impact on the HRQoL of patients with R/R CMV post-transplant.

The mean utility values for the most severe health state (CS-symptomatic CMV with lung GL) and the least severe health state (non-CS CMV) were compared with the UK EQ-5D-3L tariff [29] to contextualize the disease-specific utilities estimated for the vignettes in this study with generic utility values. CS-symptomatic CMV with lung GL had a HSUV of 0.289 which was similar to that of the 21322 EQ-5D-3L health state (0.293) [29]. This EQ-5D-3L health state suggests some problems with mobility, no problems with self-care, inability to perform usual activities, moderate pain or discomfort, and moderate anxiety or depression. Non-CS CMV had a HSUV of 0.815, similar to the utility value of the 21211 EQ-5D-3L health state (0.814). This EQ-5D-3L health state indicates some problems with mobility, no problems with self-care, some problems performing usual activities, no pain or discomfort, and no anxiety or depression. These comparisons suggest that for our study, patients would experience utility equivalent to moderate or severe problems in at least three health dimensions measured by the EQ-5D-3L.

In addition, when clinical health states were kept constant, GvHD was valued less severely than both kidney GL and lung GL, and lung GL was valued more severely than kidney GL. Thus, the extent of the impact of R/R CMV and subsequent organ loss on post-SOT HRQoL was dependent on the type of SOT received.

The background characteristics that had a statistically significant impact on the variance of health state utility values were gender, age, education level, and survey completion time. However, it should be noted that the R^2^ values were low for each health state, which suggests that these covariates are only accounting for a small proportion of variation in utility values. Therefore, it is likely that other unobserved factors influenced utility valuation. The associations of age and survey completion time on variance in health state utility values are consistent with those found in previous studies using TTOs [30]. Although a statistically significant impact of education level on health state utility values was observed, the absolute differences were marginal. Other studies have mixed findings on the association of education level with health state utility values [31, 32].

Strengths of this study include the use of multiple sources of evidence to inform the development of the vignettes, including relevant data from the existing literature and qualitative interviews with healthcare professionals experienced in treating patients with post-transplant R/R CMV. This ensured that the final vignettes accurately represented the experience of patients with post-transplant R/R CMV. The use of multiple evidence sources is also consistent with recommendations on the generation of HRQoL data outlined in a recent NICE consultation document [13]. Furthermore, the study included a large sample size that was representative of the UK general public in terms of age and sex for the online TTO survey, which is the recommended approach of valuation by NICE [33]. The application of robust quality control criteria ensured that the final set of utility data were of a high quality.

The study should, however, be considered in light of the limitation that an online, self-completion valuation approach was used rather than conducting face-to-face interviews. The decision to use this approach was driven by the impact of social restrictions caused by the COVID-19 pandemic and a desire to minimize risk of infection to participants due to COVID-19. Face-to-face interviews are the favored mode of administration for preference elicitation surveys using the TTO method. Previous studies have compared face-to-face interviewer-assisted TTO surveys with online unassisted interviews and found the data quality to be poorer in unassisted interviews [34–36]. Whilst these studies compared identical protocols based on a standard face-to-face format, our study included additional elements to inform respondents, such as an instructional video explaining the TTO process and a practice TTO task that the respondents had to complete before moving on to the main survey. Data quality was evaluated using several quality checks. A relatively high proportion of responses were excluded following the quality checks, which may be due to the choice of mode of administration, however, the sample size for analysis was still sufficient for the analysis (*n* = 738) and the analysis population is broadly representative of the UK general population for age and gender [27]. The demographic data collected in this study was limited to sex, age, education, and current health to minimize the cognitive burden on respondents since the TTO task was cognitively demanding.

## Conclusions

Health-state valuations in this study demonstrate that R/R CMV and its conventional anti-CMV treatment pose a substantial burden on post-transplant HRQoL. Although some conventional anti-CMV treatments are available for this patient population, historically these have been associated with considerable treatment-limiting toxicities, often leading to premature discontinuation of treatment, and thereby reducing effectiveness. The utility values generated in this study suggest that treatments for R/R CMV with fewer treatment-limiting toxicities could lead to large gains in health-related utility. Further, these utility values could be used in cost utility analyses of future treatments for R/R CMV. Future work could also build on the findings of this study by expanding on the patient groups included, such as those with primary CMV infection or who have received other commonly received SOTs, such as heart or liver.

### Supplementary Information

**Additional file1. Table S1.** Summary of participants' clinical experience. **Table S2.** Final vignette: Clinically significant and symptomatic CMV (CS-symptomatic CMV). **Table S3.** Final vignette: Clinically significant and symptomatic CMV (CS-symptomatic CMV) with GvHD. **Table S4.** Final vignette: Clinically significant and symptomatic CMV (CS-symptomatic CMV) with kidney graft loss. **Table S5.** Final vignette: Clinically significant and symptomatic CMV (CS-symptomatic CMV) with lung graft loss. **Table S6.** Final vignette: Clinically significant and asymptomatic CMV (CS-asymptomatic CMV). **Table S7. **Final vignette: Clinically significant and asymptomatic CMV (CS-asymptomatic CMV) with GvHD. **Table S8.** Final vignette: Clinically significant and asymptomatic CMV (CS-asymptomatic CMV) with kidney graft loss. **Table S9.** Final vignette: Clinically significant and asymptomatic CMV (CS-asymptomatic CMV) with lung graft loss. **Table S10.** Final vignette: Non-clinically significant CMV (non-CS CMV). **Table S11.** Final vignette: Non-clinically significant CMV (non-CS CMV) with GvHD. **Table S12.** Final vignette: Non-clinically significant CMV (non-CS CMV) with kidney graft loss. **Table S13.** Final vignette: Non-clinically significant CMV (non-CS CMV) with lung graft loss.

## Data Availability

The datasets, including the redacted study protocol, redacted statistical analysis plan, and individual participants data supporting the results reported in this article, will be made available within three months from initial request, to researchers who provide a methodologically sound proposal. The data will be provided after its de-identification, in compliance with applicable privacy laws, data protection and requirements for consent and anonymization.

## Abbreviations

ADHS: Adult Dental Health Survey 2009
BTEC: Business and Technician Education Council
CMV: Cytomegalovirus
CS-asymptomatic CMV: Clinically significant and asymptomatic cytomegalovirus
CS-symptomatic CMV: Clinically significant and symptomatic cytomegalovirus
GL: Graft loss
GvHD: Graft-versus-host disease
HRQoL: Health-related quality of life
HSCT: Hematopoietic stem cell transplant
HSUV: Health-state utility value
IV: Intravenous
N/A: Not available
Non-CS CMV: Non-clinically significant cytomegalovirus
NICE: National Institute for Health and Care Excellence
R/R: With or without resistance
SD: Standard deviation
SOT: Solid organ transplant
TTO: Time trade-off
