# Supplementary material for: A time trade-off study to determine health-state utilities of transplant recipients with refractory cytomegalovirus infection with or without resistance
Source: Health Qual Life Outcomes. 2024 Mar 6;22:24. doi: 10.1186/s12955-024-02239-w (PMC10919023; doi:10.1186/s12955-024-02239-w)
Supplement: Supplementary file 1 — Additional file1. Table S1. Summary of participants' clinical experience. Table S2. Final vignette: Clinically significant and symptomatic CMV (CS-symptomatic CMV). Table S3. Final vignette: Clinically significant and symptomatic CMV (CS-symptomatic CMV) with GvHD. Table S4. Final vignette: Clinically significant and symptomatic CMV (CS-symptomatic CMV) with kidney graft loss. Table S5. Final vignette: Clinically significant and symptomatic CMV (CS-symptomatic CMV) with lung graft loss. Table S6. Final vignette: Clinically significant and asymptomatic CMV (CS-asymptomatic CMV). Table S7. Final vignette: Clinically significant and asymptomatic CMV (CS-asymptomatic CMV) with GvHD. Table S8. Final vignette: Clinically significant and asymptomatic CMV (CS-asymptomatic CMV) with kidney graft loss. Table S9. Final vignette: Clinically significant and asymptomatic CMV (CS-asymptomatic CMV) with lung graft loss. Table S10. Final vignette: Non-clinically significant CMV (non-CS CMV). Table S11. Final vignette: Non-clinically significant CMV (non-CS CMV) with GvHD. Table S12. Final vignette: Non-clinically significant CMV (non-CS CMV) with kidney graft loss. Table S13. Final vignette: Non-clinically significant CMV (non-CS CMV) with lung graft loss. [file 12955_2024_2239_MOESM1_ESM.pdf]

Supplementary information

A TIME TRADE-OFF STUDY TO DETERMINE HEALTH-STATE UTILITIES OF TRANSPLANT RECIPIENTS WITH REFRACTORY CYTOMEGALOVIRUS INFECTION WITH OR WITHOUT RESISTANCE

AUTHORS

Mr Waqas Ahmed, MSc,[1*] Dr Louise Longworth PhD,[1] Dr Yemi Oluboyede PhD,[1] Mr Peter Cain, MSc,[2] Ms Stacey L. Amorosi, MA,[3] Dr Sarah Hill PhD,[1] Ms Ishan Hirji, MPH[3]

AFFILIATIONS

[1]PHMR Ltd, London, UK.

[2]Takeda UK Ltd, London, UK.

[3]Takeda Development Center Americas, Inc., Lexington, MA, USA.

*Affiliation at the time of the study.

Supplemental Figure 1. Distribution of health state utility values

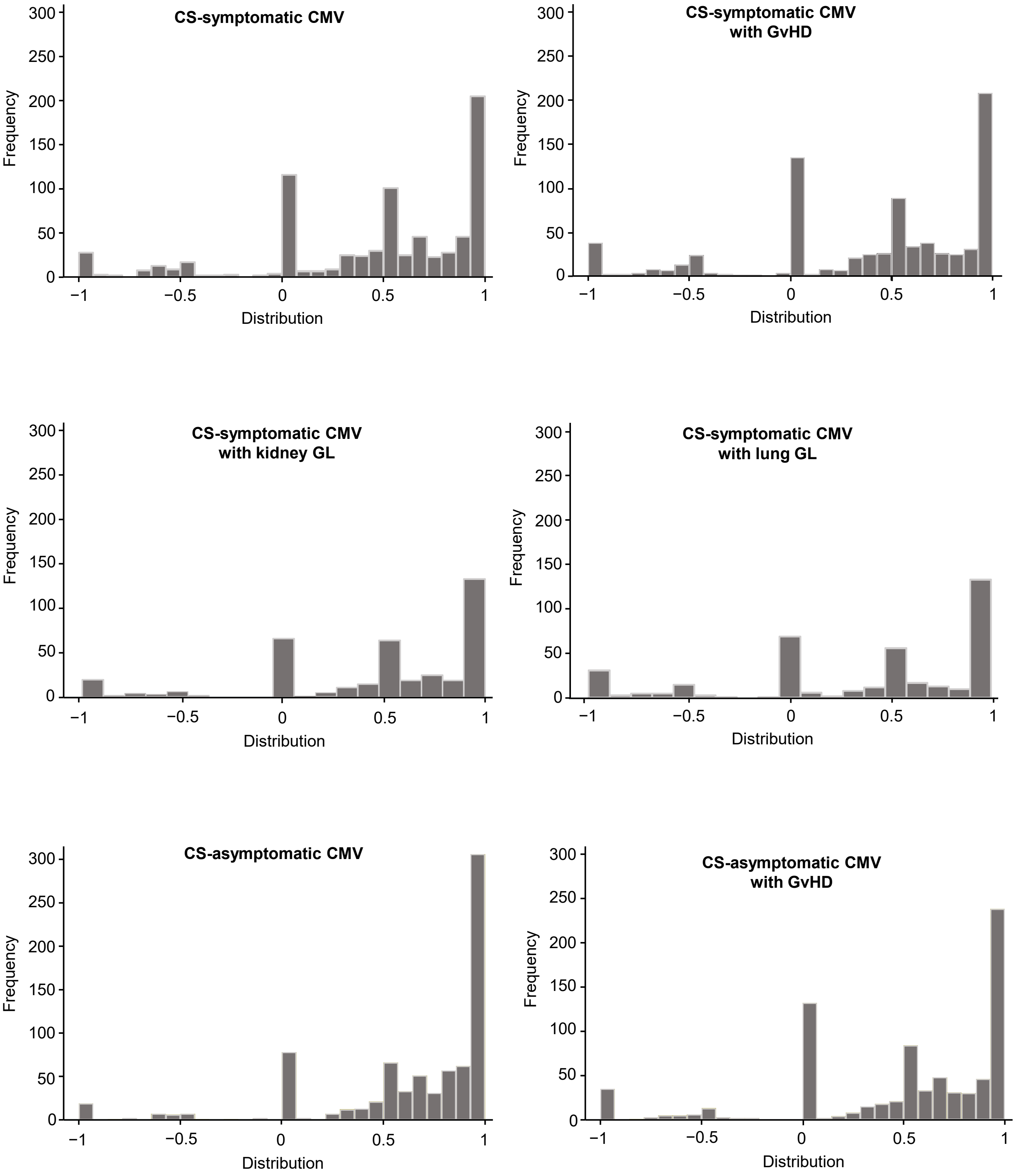

CMV, cytomegalovirus; CS-asymptomatic CMV, clinically significant and asymptomatic CMV; CS-symptomatic CMV, clinically significant and symptomatic CMV; GvHD, graft-versus-host disease; KGL, kidney graft loss; LGL, lung graft loss; non-CS CMV, non-clinically significant CMV

Supplemental Figure 1. Distribution of health state utility values (continued)

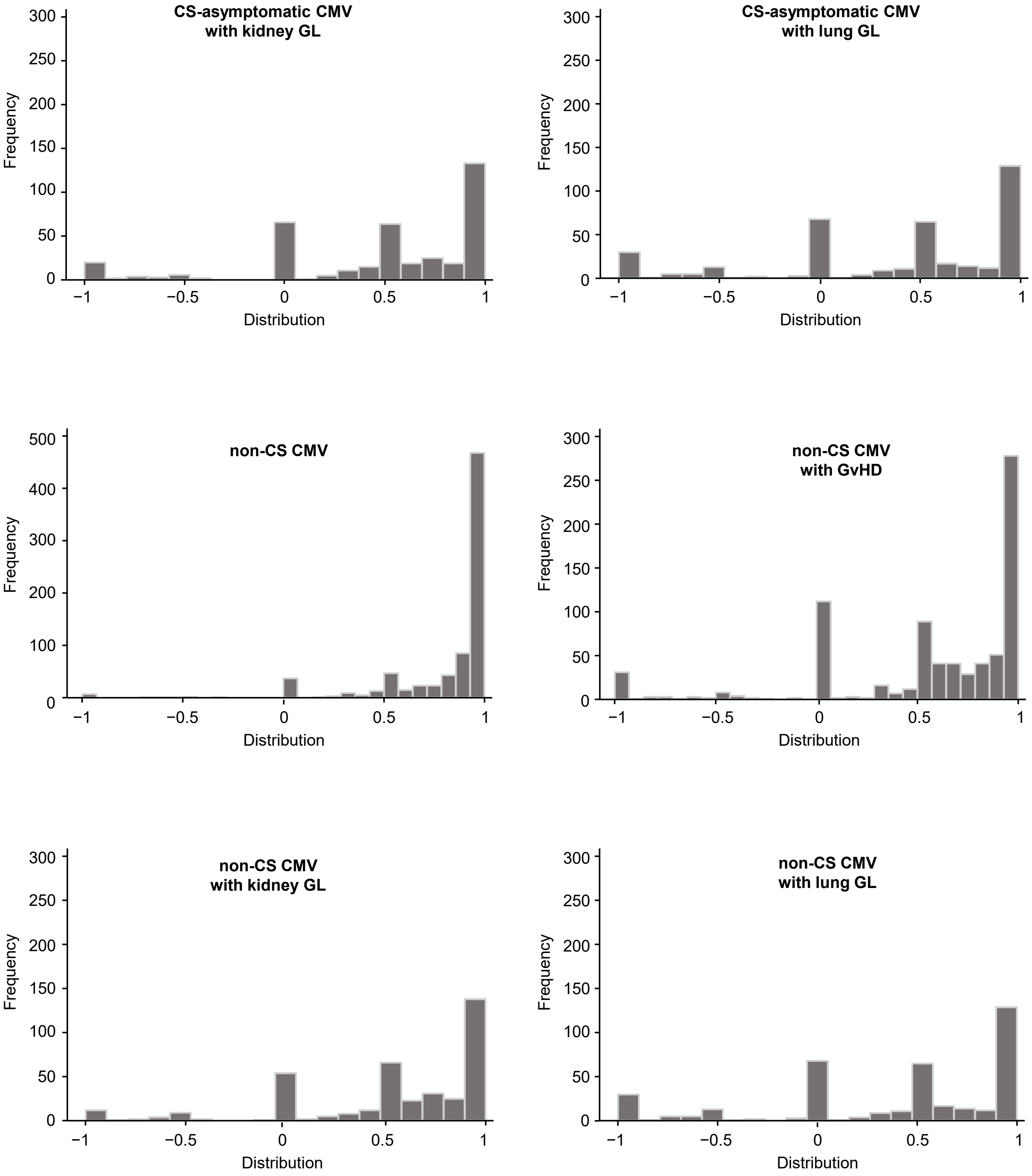

CMV, cytomegalovirus; CS-asymptomatic CMV, clinically significant and asymptomatic CMV; CS-symptomatic CMV, clinically significant and symptomatic CMV; GvHD, graft-versus-host disease; KGL, kidney graft loss; LGL, lung graft loss; non-CS CMV, non-clinically significant CMV

Table S1. Summary of participants' clinical experience

Background questions	Healthcare professionals (*N* = 5)
**Current role**	
Consultant (generic)	1
Infectious disease specialist	1
Consultant (nephrology)	1
Hematologist	2
**Type of transplant patients treated**	
Solid organ	1
Hematopoietic stem cell	2
Solid organ and stem cell	2
**Clinical experience (years)**	
0–5	0
6–10	1
11–15	3
Not reported	1

Table S2. Final vignette: Clinically significant and symptomatic CMV (CS-sCMV)

Description	• You recently received a transplant, and you now have symptoms of a virus that requires treatment.
Symptoms	• You often feel tired and lack energy. You also feel short of breath.<br>• You have loose bowel movements (diarrhea), dark urine, and dark stools. You regularly feel sick to your stomach (nausea).<br>• You have a fever, and blurred vision, and you feel generally unwell.
Treatment	• 75% of people in your situation stay in hospital for 1 to 8 weeks. They receive treatment that is delivered directly into their veins 2 to 3 times per day.<br>• The other 25% of people in your situation take oral tablets at home, twice a day for up to 3 weeks, and then once a day after this.<br>• You receive blood tests up to twice a week at the hospital.
**As a result, you experience the following:**	
Impact	• You feel depressed because of your treatment and symptoms, and you isolate yourself from others because you are worried about catching infections.<br>• You sleep and eat less because of your diarrhea and nausea, and you feel weak and embarrassed. Your diarrhea also restricts your daily activities.<br>• You need to perform exercises to regain the strength you have lost.<br>• You worry that your transplant may fail in the future.<br>• If you are staying in hospital, you have difficulty moving and are not able to work or see your family and friends as often as you would like.

Table S3. Final vignette: Clinically significant and symptomatic CMV (CS-sCMV) with GvHD

Description	• You recently received a bone marrow transplant, and you now have symptoms of a virus that requires treatment.
Symptoms	• You often feel tired and lack energy. You also feel short of breath.<br>• You have loose bowel movements (diarrhea), dark urine, and dark stools. You regularly feel sick to your stomach (nausea).<br>• You have a fever and blurred vision, and you feel generally unwell.
Treatment	• 75% of people in your situation stay in hospital for 1 to 8 weeks. They receive treatment that is delivered directly into their veins 2 to 3 times per day.<br>• The other 25% of people in your situation take oral tablets twice a day for up to 3 weeks, and then once a day after this.<br>• You receive blood tests up to twice a week at the hospital.
**In addition to the above, you also experience the following:**	
Description	• Your recent bone marrow transplant has reacted to your body, and you have some symptoms because of this. You need treatment for this reaction.
Symptoms	• You have developed an itchy, red skin rash that sometimes hurts.<br>• Your skin and eyes look yellow, and your urine and stool are darker than normal.<br>• You have stomach pain, stomach cramps, and loose bowel movements (diarrhea). You feel sick to your stomach (nausea) and are vomiting.
Treatment	• You have tests in the hospital to see if your transplant has reacted to your body.<br>• You are regularly checked to see if you are dehydrated because of diarrhea and vomiting.<br>• You stay in hospital and receive steroid treatment that is delivered directly into your veins daily.
**As a result, you experience the following:**	
Impact	• You sleep and eat less because of your diarrhea and nausea, and you feel weak and embarrassed. Your diarrhea also restricts your daily activities.<br>• You need to perform exercises to regain the strength you have lost.<br>• Because of your treatment and symptoms, you have difficulty moving. You are isolated from others because you are at risk of developing other infections.<br>• You worry that your transplant is failing because of how it is reacting to your body.

Table S4. Final vignette: Clinically significant and symptomatic CMV (CS-sCMV) with kidney graft loss

Description	• You recently received a kidney transplant, and you now have symptoms of a virus that requires treatment.
Symptoms	• You often feel tired and lack energy. You also feel short of breath.<br>• You have loose bowel movements (diarrhea), dark urine, and dark stools. You regularly feel sick to your stomach (nausea).<br>• You have a fever, blurred vision, and you feel generally unwell.
Treatment	• 75% of people in your situation stay in hospital for 1 to 8 weeks. They receive treatment that is delivered directly into their veins 2 to 3 times per day.<br>• The other 25% of people in your situation take oral tablets at home, twice a day for up to 3 weeks, and then once a day after this.<br>• You receive blood tests up to twice a week at the hospital.
**In addition to the above, you also experience the following:**	
Description	• Hospital tests confirm that your kidney transplant has now failed.<br>• You need to be monitored by healthcare professionals.
Symptoms	• You have swelling and pain over the area where your kidneys are.<br>• You also have trouble passing urine.
Treatment	• You require 2 to 3 dialysis treatment sessions per week in a hospital, each lasting between 3 and 5 hours. This involves your blood being taken from your body by a tube, filtered through a machine and returned to your body via a tube.<br>• This treatment will be required until you are able to have another kidney transplant.
**As a result, you experience the following:**	
Impact	• You feel depressed because of your treatment and symptoms, and you isolate yourself from others because you are worried about catching infections.<br>• You sleep and eat less because of your diarrhea and nausea, and you feel weak and embarrassed. Your diarrhea and dialysis also restrict your daily activities.<br>• You need to perform exercises to regain the strength you have lost.

	• Because of your dialysis, you have nausea, dizziness, and muscle cramps during treatment, as well as weight loss and difficulties falling asleep.<br>• You worry if you will be able to receive another kidney transplant in the future, and how long you will have to wait for a future transplant.<br>• If you are staying in hospital, you have difficulty moving and are not able to work or see your family and friends as often as you would like.

Table S5. Final vignette: Clinically significant and symptomatic CMV (CS-sCMV) with lung graft loss

Description	• You recently received a lung transplant, and you now have symptoms of a virus that requires treatment.
Symptoms	• You often feel tired and lack energy. You also feel short of breath.<br>• You have loose bowel movements (diarrhea), dark urine, and dark stools. You regularly feel sick to your stomach (nausea).<br>• You have a fever, blurred vision, and you feel generally unwell.
Treatment	• 75% of people in your situation stay in hospital for 1 to 8 weeks. They receive treatment that is delivered directly into their veins 2 to 3 times per day.<br>• The other 25% of people in your situation take oral tablets twice a day for up to 3 weeks, and then once a day after this.<br>• You receive blood tests up to twice a week at the hospital.
**In addition to the above, you also experience the following:**	
Description	• Hospital tests confirm that your lung transplant has now failed.<br>• You need to be monitored by healthcare professionals.
Symptoms	• You are short of breath and have a dry cough.<br>• You feel extremely tired and confused, and you find it difficult to stay awake.
Treatment	• You are attached to an intubation machine. This means a tube has been put down your throat and into your windpipe, with air pumped down the tube and into your lungs.<br>• You need to be looked after daily.
**As a result, you experience the following:**	
Impact	• You feel depressed because of your treatment and symptoms, and you isolate yourself from others because you are worried about catching infections.<br>• You sleep and eat less because of your diarrhea and nausea, and you feel weak and embarrassed. Your diarrhea also restricts your daily activities.<br>• You need to perform exercises to regain the strength you have lost.<br>• Because you are intubated, your throat is sore and painful, and you are not able to speak. You are not able to eat or drink, and you are now given food and medication through a tube.

	• You are limited in your movement, and you are not able to see your family and friends as often as you would like.<br>• You have been placed back on the waiting list for another transplant. You are worried that another suitable organ will not become available.

Table S6. Final vignette: Clinically significant and asymptomatic CMV (CS-aCMV)

Description	• You recently received a transplant, and you have a virus that requires treatment.<br>• You do not currently have any symptoms from your virus, but you may experience symptoms later.
Symptoms	• You are worried that your condition will get worse, and cause symptoms.<br>• These symptoms might include feeling sick to your stomach (nausea), fever, feeling unwell and tired, blurred vision, shortness of breath, loose bowel movements (diarrhea), dark urine, and dark stools.
Treatment	• You take a tablet at home to treat the virus. This tablet is taken twice a day for up to 3 weeks, and then once a day after this.<br>• You receive blood tests up to twice a week at the hospital.
**As a result, you experience the following:**	
Impact	• You feel sick to your stomach (nausea), feel tired and short of breath, and experience diarrhea because of your medication.<br>• You are frustrated that you must take medication, even though you do not currently have any symptoms from your virus.<br>• Your worry that the symptoms of your virus may return and that your transplant may fail in the future.

Table S7. Final vignette: Clinically significant and asymptomatic CMV (CS-aCMV) with GvHD

Description	• You recently received a bone marrow transplant, and you have a virus that requires treatment.<br>• You do not currently have any symptoms from your virus, but you may experience symptoms later.
Symptoms	• You are worried that your condition will get worse, and cause symptoms.<br>• These symptoms might include fever, feeling unwell and tired, blurred vision, and shortness of breath.
Treatment	• You take a tablet to treat the virus. This tablet is taken twice a day for up to 3 weeks, and then once a day after this.<br>• You receive blood tests up to twice a week at the hospital.
**In addition to the above, you also experience the following:**	
Description	• Your recent bone marrow transplant has reacted to your body, and you have some symptoms because of this. You need treatment for this reaction.
Symptoms	• You have developed an itchy, red skin rash that sometimes hurts.<br>• Your skin and eyes look yellow, and your urine and stool are darker than normal.<br>• You have stomach pain, stomach cramps, and loose bowel movements (diarrhea). You feel sick to your stomach (nausea) and are vomiting.
Treatment	• You have tests in the hospital to see if your transplant has reacted to your body.<br>• You are regularly checked to see if you are dehydrated because of diarrhea and vomiting.<br>• You stay in hospital and receive steroid treatment that is delivered directly into your veins daily.
**As a result, you experience the following:**	
Impact	• Because of your medication, you feel sick to your stomach (nausea), feel tired and short of breath, and experience diarrhea.<br>• You are frustrated that you must take medication, even though you do not currently have any symptoms from your virus.<br>• You sleep and eat less because of your diarrhea and nausea, and you feel weak and embarrassed. Your diarrhea also restricts your daily activities.<br>• You need to perform exercises to regain the strength you have lost.<br>• Because of your treatment and symptoms, you have difficulty moving. You are isolated from others because you are at risk of developing other infections.<br>• You worry that your transplant is failing because of how it is reacting to your body, and that the symptoms of your virus may return in the future.

Table S8. Final vignette: Clinically significant and asymptomatic CMV (CS-aCMV) with kidney graft loss

Description	• You recently received a kidney transplant, and you have a virus that requires treatment.<br>• You do not currently have any symptoms from your virus, but you may experience symptoms later.
Symptoms	• You are worried that your condition will get worse, and cause symptoms.<br>• These symptoms might include feeling sick to your stomach (nausea), fever, feeling unwell and tired, blurred vision, shortness of breath, loose bowel movements (diarrhea), dark urine, and dark stools.
Treatment	• You take a tablet at home to treat the virus. This tablet is taken twice a day for up to 3 weeks, and then once a day after this.<br>• You receive blood tests up to twice a week at the hospital.
**In addition to the above, you also experience the following:**	
Description	• Hospital tests confirm that your kidney transplant has now failed.<br>• You need to be monitored by healthcare professionals.
Symptoms	• You have swelling and pain over the area where your kidneys are.<br>• You have trouble passing urine.
Treatment	• You require 2 to 3 dialysis treatment sessions per week in a hospital, each lasting between 3 and 5 hours. This involves your blood being taken from your body by a tube, filtered through a machine, and returned to your body via a tube.<br>• This treatment will be required until you are able to have another kidney transplant.
**As a result, you experience the following:**	
Impact	• Because of your medication, you feel sick to your stomach (nausea), feel tired and short of breath, and experience diarrhea.<br>• You are frustrated that you must take medication, even though you do not currently have any symptoms from your virus.<br>• Because of your dialysis, you have nausea, dizziness, and muscle cramps during treatment, as well as weight loss and difficulties falling asleep. Your dialysis also restricts your daily activities.<br>• You are worried about if you will be able to receive another kidney transplant in the future, and how long you will have to wait for a future transplant. You are also worried that the symptoms of your virus may return in the future.

Table S9. Final vignette: Clinically significant and asymptomatic CMV (CS-aCMV) with lung graft loss

Description	• You recently received a lung transplant, and you have a virus that requires treatment.<br>• You do not currently have any symptoms from your virus, but you may experience symptoms later.
Symptoms	• You are worried that your condition will get worse, and cause symptoms.<br>• These symptoms might include feeling sick to your stomach (nausea), fever, feeling unwell, blurred vision, loose bowel movements (diarrhea), dark urine, and dark stools.
Treatment	• You take a tablet to treat the virus. This tablet is taken twice a day for up to 3 weeks, and then once a day after this.<br>• You receive blood tests up to twice a week at the hospital.
**In addition to the above, you also experience the following:**	
Description	• Hospital tests confirm that your lung transplant has now failed.<br>• You need to be monitored by healthcare professionals.
Symptoms	• You are short of breath and have a dry cough.<br>• You feel extremely tired and confused, and you find it difficult to stay awake.
Treatment	• You are attached to an intubation machine. This means a tube has been put down your throat and into your windpipe, with air pumped down the tube and into your lungs.<br>• You need to be looked after daily.
**As a result, you experience the following:**	
Impact	• Because of your medication, you feel sick to your stomach (nausea), feel tired and short of breath, and experience diarrhea.<br>• You are frustrated that you must take medication, even though you do not currently have any symptoms from your virus.<br>• Because you are intubated, your throat is sore and painful, and you are not able to speak. You are not able to eat or drink, and you are now fed food and medication through a tube.<br>• You are limited in your movement, and you are not able to see your family and friends as often as you would like.<br>• You have been placed back on the waiting list for another transplant. You are worried that another suitable organ will not become available. You are also worried that the symptoms of your virus may return in the future.

Table S10. Final vignette: Non-clinically significant CMV (N-csCMV)

Description	• You recently received a transplant, and you developed a virus that was treated. The virus is not currently active and does not currently require treatment.<br>• You do not currently have any symptoms from your virus, but you may experience symptoms later if the virus becomes active again.
Symptoms	• You are worried that your virus will become active again and cause symptoms.<br>• These symptoms might include feeling sick to your stomach (nausea), fever, blurred vision, feeling unwell and tired, feeling short of breath, loose bowel movements (diarrhea), dark urine, and dark stools.
Treatment	• You are worried that you may need treatment for your virus again.<br>• You also receive blood tests up to twice a week at the hospital to make sure your virus has not returned.
**As a result, you experience the following:**	
Impact	• You are worried about developing viral symptoms and needing treatment again. However, the virus currently has no other impact on your life.

Table S11. Final vignette: Non-clinically significant CMV (N-csCMV) with GvHD

Description	• You recently received a bone marrow transplant, and you developed a virus that was treated. The virus is not currently active and does not currently require treatment.<br>• You do not currently have any symptoms from your virus, but you may experience symptoms later if the virus becomes active again.
Symptoms	• You are worried that your virus will become active again and cause symptoms.<br>• These symptoms might include a fever, blurred vision, feeling unwell and tired, and feeling short of breath.
Treatment	• You are worried that you may need treatment for your virus again.<br>• You also receive blood tests up to twice a week at the hospital to make sure your virus has not returned.
**In addition to the above, you also experience the following:**	
Description	• Your recent bone marrow transplant has reacted to your body, and you have some symptoms because of this. You need treatment for this reaction.
Symptoms	• You have developed an itchy, red skin rash that sometimes hurts.<br>• Your skin and eyes look yellow, and your urine and stool are darker than normal.<br>• You have stomach pain, stomach cramps, and loose bowel movements (diarrhea). You feel sick to your stomach (nausea) and are vomiting.
Treatment	• You have tests in the hospital to see if your transplant has reacted to your body.<br>• You are regularly checked to see if you are dehydrated because of diarrhea and vomiting.<br>• You stay in hospital and receive steroid treatment that is delivered directly into your veins daily.
**As a result, you experience the following:**	
Impact	• You sleep and eat less because of your diarrhea and nausea, and you feel weak and embarrassed. Your diarrhea also restricts your daily activities.<br>• You need to perform exercises to regain the strength you have lost.<br>• Because of your treatment and symptoms, you have difficulty moving. You are isolated from others because you are at risk of developing other infections.<br>• You worry that your transplant is failing because of how it is reacting to your body.<br>• You are also worried about developing viral symptoms and needing treatment again. However, the virus currently has no other impact on your life.

Table S12. Final vignette: Non-clinically significant CMV (N-csCMV) with kidney graft loss

Description	• You recently received a kidney transplant, and you developed a virus that was treated. The virus is not currently active and does not currently require treatment.<br>• You do not currently have any symptoms from your virus, but you may experience symptoms later if the virus becomes active again.
Symptoms	• You are worried that your virus will become active again and cause symptoms.<br>• These symptoms might include feeling sick to your stomach (nausea), fever, blurred vision, feeling unwell and tired, feeling short of breath, loose bowel movements (diarrhea), dark urine, and dark stools.
Treatment	• You receive blood tests up to twice a week at the hospital to make sure your virus has not returned.
**In addition to the above, you also experience the following:**	
Description	• Hospital tests confirm that your kidney transplant has now failed.<br>• You need to be monitored by healthcare professionals.
Symptoms	• You have swelling and pain over the area where your kidneys are.<br>• You have trouble passing urine.
Treatment	• You require 2 to 3 dialysis treatment sessions per week in a hospital, each lasting between 3 and 5 hours. This involves your blood being taken from your body by a tube, filtered through a machine and returned to your body via a tube.<br>• This treatment will be required until you are able to have another kidney transplant.
**As a result, you experience the following:**	
Impact	• Because of your dialysis, you have nausea, dizziness, and muscle cramps during treatment, as well as weight loss and difficulties falling asleep. Your dialysis also restricts your daily activities.<br>• You worry if you will be able to receive another kidney transplant in the future, and how long you will have to wait for a future transplant.<br>• You are also worried about developing viral symptoms and needing treatment again. However, the virus currently has no other impact on your life.

Table S13. Final vignette: Non-clinically significant CMV (N-csCMV) with lung graft loss

Description	• You recently received a lung transplant, and you developed a virus that was treated. The virus is not currently active and does not currently require treatment.<br>• You do not currently have any symptoms from your virus, but you may experience symptoms later if the virus becomes active again.
Symptoms	• You are worried that your virus will become active again and cause symptoms.<br>• These symptoms might include feeling sick to your stomach (nausea), fever, blurred vision, feeling unwell, loose bowel movements (diarrhea), dark urine, and dark stools.
Treatment	• You receive blood tests up to twice a week at the hospital to make sure your virus has not returned.
**In addition to the above, you also experience the following:**	
Description	• Hospital tests confirm that your lung transplant has now failed.<br>• You need to be monitored by healthcare professionals.
Symptoms	• You are short of breath and have a dry cough.<br>• You feel extremely tired and confused, and find it difficult to stay awake.
Treatment	• You are attached to an intubation machine. This means a tube has been put down your throat and into your windpipe, with air pumped down the tube and into your lungs.<br>• You need to be looked after daily.
**As a result, you experience the following:**	
Impact	• Because you are intubated, your throat is sore and painful, and you are not able to speak. You are not able to eat or drink, and you are now given food and medication through a tube.<br>• You are limited in your movement, and you are not able to see your family and friends as often as you would like.<br>• You have been placed back on the waiting list for another transplant. You are worried that another suitable organ will not become available.<br>• You are also worried about developing viral symptoms and needing treatment again. However, the virus currently has no other impact on your life.
